# A pilot study: Can calcaneus radiographic image be used to determine sex and breed in cats?

**DOI:** 10.1002/vms3.899

**Published:** 2022-08-03

**Authors:** Esra Şenol, Ozan Gündemir, Sokol Duro, Tomasz Szara, Yasin Demiraslan, Hüseyin Karadağ

**Affiliations:** ^1^ Institute of Graduate Studies Istanbul University‐Cerrahpasa Istanbul Turkey; ^2^ Faculty of Veterinary Medicine, Department of Anatomy Istanbul University‐Cerrahpasa Istanbul Turkey; ^3^ Faculty of Veterinary Medicine Agricultural University of Tirana Tirana Albania; ^4^ Department of Morphological Sciences Institute of Veterinary Medicine Warsaw University of Life Sciences Warsaw Warszawa Poland; ^5^ Faculty of Veterinary Medicine Department of Anatomy Burdur Mehmet Akif Ersoy University Burdur Turkey; ^6^ Faculty of Dentistry Department of Basic Sciences Istanbul Gelisim University Istanbul Turkey

**Keywords:** cat, calcaneus, radiometry, radiographic images, tarsus

## Abstract

This study examined whether radiographic images measurements of the calcaneus in cats are determinative of sex and breed. For this purpose, radiographic images of 70 cats (37 male and 33 females) of different ages (from one to 18 years) and different breeds (41 mix‐breed, 18 Scottish Fold and 11 British Shorthair cats) without orthopaedic problems were used. Right tarsal joint radiographs of these orthopaedically healthy cats were taken. Four linear measurements and two angle values of the calcaneus were obtained from the radiographic images. The MANOVA result showed that the most determining factor between the three groups was the greatest width (*p* value = 0.001). Calcaneal body length, calcaneal greatest length and calcaneal shortest depth were higher in mix‐breed cats. Calcaneal tuber length was higher in Scottish Fold cats. The only statistically significant difference between Scottish Fold and British Shorthair was in the calcaneal tuber length (*p* value = 0.04). In the comparison made between the sexes regardless of species, the linear measurements in males were higher than in females. It was determined that these parameters are statistically significant in terms of sex differentiation in cats. Dorsal and plantar calcaneal angles are not sex determinants in cats. The effect of age on other measurements was analysed by correlation test. However, the effect of age on the measurements was not statistically significant. Mix‐breed cats were examined in four groups according to their colour (grey, black‐white, yellow, tri‐colour). No statistically significant difference was found between calcaneal measurements of cats with different skin colour genotypes. In this study, calcaneus measurements were both determinative between breeds and sexes in cats.

## Graphical abstract

Right tarsal joint radiographic images of 70 cats (37 male and 33 females) of different ages (from one to 18 years) and different breeds (41 mix‐breed, 18 Scottish Fold and 11 British Shorthair cats) without orthopedic problems were used. The MANOVA result showed that the most determining factor between the three groups was the greatest width (P value: 0.001). In the comparison made between the sexes regardless of species, the linear measurements in males were higher than in females. In this study, calcaneus measurements were both determinative between breeds and sexes in cats.

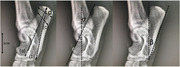

## 1 INTRODUCTION

The domestic cat (*Felis catus*, Linneas 1758) was domesticated approximately 9000–10,000 years ago (Vigne et al., 2004). During domestication, instead of following herds of wildlife, cats gradually adopted a rural lifestyle with humans (Gupta, 2004). Of the 41 breeds recognised by the Cat Fanciers' Association (CFA), 16 are designated as ‘natural’ breeds, and the remaining breeds have developed in the last 50 years (Lipinski et al., 2008). Scottish Fold and British Shorthair have been recognised as a separate breed by The International Cat Association (https://www.tica.org/breeds/browse‐all‐breeds). Most of the breeds encountered on the streets are considered mix‐breed cats, although they resemble their ancestor (*Felis silvestris lybica*) (Driscoll et al., 2007). The fact that these cats are yellow or black is due to the intensity of the pigments that give colour to their coat conditioned by the dominant gene pair (Schmidt‐Küntzel et al., 2009).

Dogs and cats support themselves by the digits only and thus are classified as intermediate stage or the digitigrade posture animals compared to the ungulate animals that are classified in the unguligrade posture in which only the tips of the digits, protected by hooves, give support (Dyce et al., 2017; Nechiporuk et al., 2020).

Information on the morphological structure of pelvic limb helps to understand the lever function and forward thrust mechanics while performing movements such as hunting or climbing. Ankle morphology has also proven to be significantly associated with movement and posture (Polly, 2008). The tarsal and metatarsal bones are nearly vertical in digitigrade type and, the stance positions of the tarsal joints in carnivores differ from unguligrade such as cows and horses (Kimura, 1996). Cats can be called carnivorous species that spend a lot of time on the ground but are also skilled climbers (Taylor, 1989).

Tarsal joints have high mobility, including extension and flexion (Voss et al., 2004). The cat's tarsus has seven bones arranged in three rows, with the talus and calcaneus being the largest bones and located in the proximal row (Constantinescu, 2018; Getty, 1975; König & Liebich, 2020; Nickel et al., 1986). The tarsal region is clinically important for cats because tarsal joint injuries in cats are very common, especially caused by road traffic accidents, falls from heights or dog bites (Kulendra & Arthurs, 2014; Polat et al., 2017). Owen (2000) and Rochlitz (2003) in their studies conclude that tarsal injuries in male cats are overrepresented compared to female cats because tomcats have a wide home range and roam in search of oestrus females.

Osteometric differences between males and females, as well as differences between species have been examined directly in the skeletal bones of the limbs as well as in the skull bones of various animals in studies conducted by Gündemir et al. (2020), Kobryńczuk et al. (2008), Nganvongpanit et al. (2017) and Szara et al. (2003) but also from studies performed with measurements according to radiographic images (Eneroth et al., 1999; Gündemir et al., 2021; Limmanont et al., 2019; Oheida et al., 2019; Uzuner et al., 2016).

In some studies, the measurements of the appendicular skeleton were taken and the correlations with the age and height of the animal were established (Bakhtiari & Heshmat, 2009; Onar et al., 2018). In other studies, conducted in humans, it has been observed that radiometric measurements of calcaneus play a decisive role in sex determination (Bidmos & Asala, 2003; Riepert et al., 1996; Uzuner et al., 2016). Also, the study conducted by Asala (2001) shows that bone measurements performed in humans are specific to different populations.

Calcaneus is a strong bone and easily identifiable due to its characteristic shape and strong protective properties. The calcaneus has a solid projection, which the common tendon of the calcaneus, formed by the gastrocnemius and soleus muscles, is attached. Therefore, these structures constitute the internal component of the strong plantar flexion required by the hind leg in biomechanical functions such as jumping, pushing the body forward and climbing (Polly, 2010). In this case, the question of whether there is any difference in the morphological features of the structures participating in the function of the hind limbs in domestic cats compared to wild cats that maintain their hunting ability without interruption, seems meaningful.

The calcaneus is the largest bone of the tarsus. It was observed that calcaneus kept its shape for a long time in cadavers, mummified or even in the archaeological findings. It is thought that it will be important to use linear and angular measurements taken such as a sample, which can maintain its shape for a long time, in sex determination. From the literature observations, it has been not found any study that has performed measurements of radiographic images of feline calcaneus, so comparative data are missing.

The aim of this pilot study was to investigate whether calcaneal measurements in radiological images as used in humans can be sex or species determining in cats. In addition, calcaneus measurements were also examined in mixed‐breed cats of different colours (grey, black‐white, yellow, tricolour).

## 2 MATERIALS AND METHODS

### 2.1 Cats and radiographic images

The study was performed on radiographic images of 70 cats (37 male and 33 females) of different ages (from one to 18 years) and different breeds (41 mix‐breed, 18 Scottish Fold and 11 British Shorthair cats) without orthopaedic problems. X‐ray images of cats were taken with the permission of their owners. Fujifilm FCR prima t2 model X‐ray machine was used. Images were obtained with an X‐ray device from 30 centimetres. Mediolateral radiographs were taken only from the right tarsal region of the cats. Measurements of the images were taken in the Radiant DICOM program in centimetre unit (cm). In order to carry out the study, permission Nr. 60618/16.03.2021 was obtained from the Ethics Committee of Istanbul University‐Cerrahpaşa, Faculty of Veterinary Medicine.

### 2.2 Radiometric measurements

**FIGURE 1:**
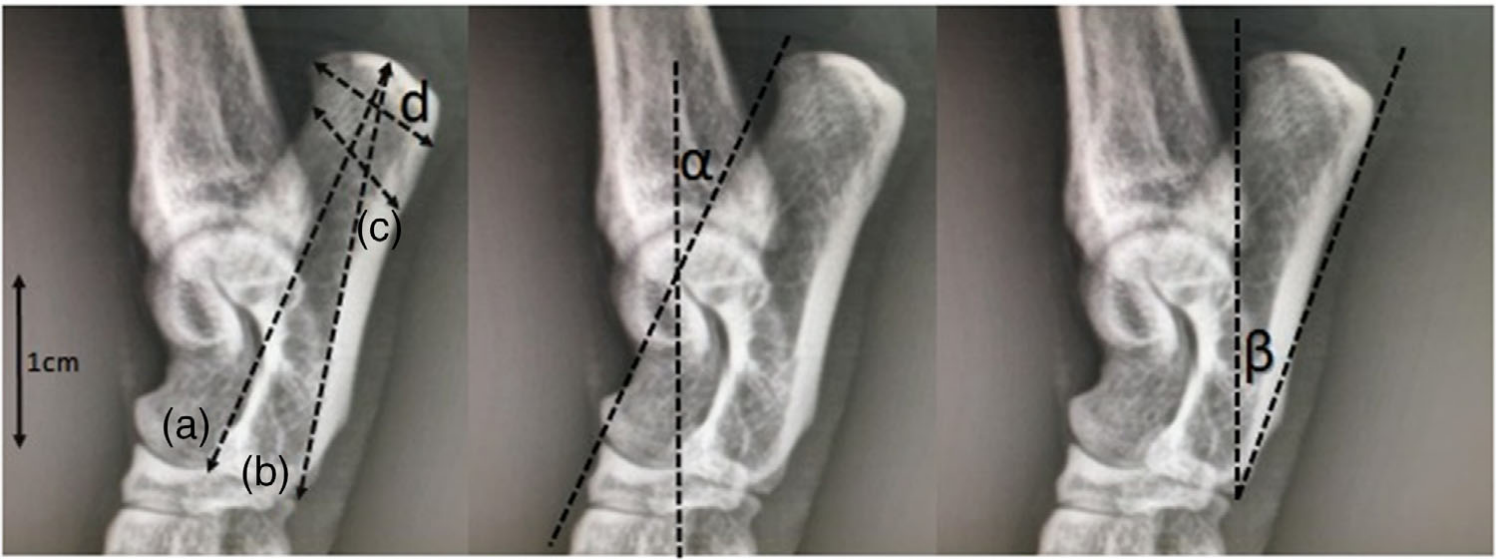
The linear and angles radiometric measurements of cat calcaneus, calcaneal body length (*a*), calcaneal greatest length (*b*), calcaneal shortest depth (*c*), calcaneal tuber length (*d*), dorsal calcaneal angle (*α*) and plantar calcaneal angle (*β*).

Four liner and two angles radiometric measurements of cat calcaneus (Figure 1) were performed on radiographic images of the right tarsal region of cats based on the study conducted in humans by Uzuner et al. (2016) and Kim et al. (2013) as below:

**Calcaneal body length (CBL)**: The distance in between the most proximal point of calcaneus tuberosity and the most dorsal‐mediodistal point of the calcaneal base.

**Calcaneal greatest length** (**CGL**): The distance in between the most proximal point of calcaneus tuberosity and the most plantar‐mediodistal point of the calcaneal base.

**Calcaneal shortest depth** (**CSD**): The shortest distance between cranial and caudal surface of the calcaneal body.

**Calcaneal tuber length** (**CTL**): The distance in between the most dorsal and plantar points of the calcaneal tuberosity.

**Dorsal calcaneal angle** (**DCA**): The angle between the line drawn from most dorsal point of calcaneal tuberosity to the coracoid process and the line drawn in the direction from the coracoid process to the most dorsal‐mediodistal point of the calcaneal base.

**Plantar calcaneal angle (PCA)**: The angle created at the most plantar‐mediodistal point of the calcaneal base with the line going in direction to the most dorsal and plantar points of the calcaneal tuberosity.

### 2.3 Statistical analysis

SPSS 22 package program was used for statistical analysis. The mean values, standard deviations and *p* values of the results were obtained and shown in the Tables 1, 2, 3, 4. Three groups were created to compare cat breeds (mix‐breed, Scottish Fold and British Shorthair). The MANOVA test was used to determine the most predictive measure among these three groups. Using the ANOVA test, it was examined whether there were statistical differences between the sexes. ANOVA was also used for pairwise comparison between cat breeds. Mixed breed cats were examined in four groups according to their colour (grey, black‐white, yellow, tricolour) and the statistical difference between them was analysed by MANOVA. Normality assumption was checked by Levene's test. The correlations of the results with each other were examined. Statistical difference was accepted as *p* < 0.05.

**TABLE 1:** Means, standard deviations and *p* values of radiometric measurements of breeds (MANOVA)

Parameters	Mix‐breed *N*: 41	Scottish Fold *N*: 18	British Shorthair *N*: 11	*p* Value
Age	4.79 ± 3.59	2.81 ± 1.42	3.68 ± 1.94	‐
Calcaneal body length (cm)	2.75 ± 0.2	2.56 ± 0.2	2.58 ± 0.17	0.001
Calcaneal greatest length (cm)	2.83 ± 0.21	2.65 ± 0.21	2.68 ± 0.13	0.004
Calcaneal shortest depth (cm)	0.76 ± 0.08	0.75 ± 0.08	0.74 ± 0.08	0.608
Calcaneal tuber length (cm)	0.82 ± 0.09	0.87 ± 0.11	0.78 ± 0.09	0.071
Dorsal calcaneal angle (°)	18.22 ± 1.97	20.01 ± 2.42	18.6 ± 2.08	0.014
Plantar calcaneal angle (°)	18.38 ± 4.18	17.96 ± 5.46	18.89 ± 3.8	0.861

**TABLE 2:** *p* Values as a result of pairwise comparison between groups (ANOVA)

Parameters	Scottish Fold British Shorthair	Mix‐breed British Shorthair	Mix‐breed Scottish Fold
Calcaneal body length	0.723	0.014	0.001
Calcaneal greatest length	0.659	0.030	0.004
Calcaneal shortest depth	0.763	0.378	0.509
Calcaneal tuber length	0.045	0.197	0.113
Dorsal calcaneal angle	0.121	0.573	0.004
Plantar calcaneal angle	0.623	0.716	0.745

**TABLE 3:** Linear measurements and angle values of the calcaneus between cat sex (ANOVA)

Parameters	Sex	*N*	Mean	SD	Minimum	Maximum	*F*	*p* Value
Calcaneal body length (cm)	Male	37	2.76	0.19	2.34	3.20	16.13	0.00
	Female	33	2.58	0.19	2.15	3.01		
Calcaneal greatest length (cm)	Male	37	2.86	0.19	2.49	3.21	23.76	0.00
	Female	33	2.64	0.18	2.33	3.13		
Calcaneal shortest depth (cm)	Male	37	0.79	0.07	0.63	0.95	21.82	0.00
	Female	33	0.72	0.07	0.56	0.84		
Calcaneal tuber length (cm)	Male	37	0.87	0.11	0.69	1.11	14.39	0.00
	Female	33	0.78	0.07	0.65	0.95		
Dorsal calcaneal angle (°)	Male	37	18.79	2.27	14.70	24.60	0.04	0.83
	Female	33	18.68	2.18	14.20	23.90		
Plantar calcaneal angle (°)	Male	37	18.89	4.55	9.20	31.10	1.18	0.28
	Female	33	17.74	4.28	9.20	27.30		

**TABLE 4:** Correlation coefficients between measurements (without breed or sex discrimination)

Variables	*a*	*b*	*c*	*d*	*α*	*β*
Age	–0.079	–0.039	0.030	–0.043	0.027	–0.020
*a*		0.933**	0.632**	0.458**	–0.193	0.007
*b*			0.570**	0.416**	–0.246*	–0.086
*c*				0.830**	0.477**	–0.077
*d*					0.682**	–0.232
*α*						–0.185

** Correlation is significant at the 0.01 level (2‐tailed).

* Correlation is significant at the 0.05 level (2‐tailed).

*Note*: Calcaneal body length (*a*), calcaneal greatest length (*b*), calcaneal shortest depth (*c*), calcaneal tuber length (*d*), dorsal calcaneal angle (*α*), plantar calcaneal angle (*β*).

## 3 RESULTS

In Table 1, radiometric measurement values between cat groups (mix‐breed, Scottish Fold and British Shorthair) were analysed regardless of sex. MANOVA was performed for differences between groups. The most determining factor between the groups was the CBL (*p* value = 0.001). CBL, CGL and CSD values were higher in mix‐breed cats.

ANOVA test was used to compare paired groups (Table 2). CTL measurement was higher in Scottish Fold cats. The only statistically significant difference between Scottish Fold and British Shorthair was in the CTL (*p* value = 0.04).

Table 3 shows radiometric measurements related to cat sex determination. In the comparison made between the sexes regardless of species, the linear measurements of the males were higher than the females (*p* < 0.001). It was determined that PCA and DCA were not determinative in terms of sex.

The correlation values between the measurements are given in Table 4. Age and PCA have no effect on other measurements. The highest correlation value was between CBL and CGL measurements.

Mix‐breed cats were examined in four groups according to their colour (grey, black‐white, yellow, tri‐colour). All measurements of grey coloured cats were higher than other mix‐breed cats. However, no statistically significant difference was found between calcaneal measurements of cats with different skin colour genotypes (MANOVA). No statistical difference was found in paired group comparisons either.

## 4 DISCUSSION

The linear measurements of radiographic images of the calcaneus were quite determinant between male and female cats, where the measurement values of male individuals were higher than females for all breed groups used in the study. Angle measurements were not as effective in sex determination as linear measurements. In this study it was observed that age of cats had no effect on the measurements, although a wide range of cat ages were included.

In literature there are many studies showings that the results obtained using radiographic images of the calcaneus are determinant in the differentiation between males and females. Uzuner et al. (2016) conducted a study on 143 people and reported that all calcaneal radiograph measurements used in this study were statistically determinative between males and females. Bidmos and Dayal (2003) also reported that linear measurements of tarsal bone in male individuals are higher than females in their study.

The study showed that linear measurements performed on radiographic images of cat's calcaneus are a simple, effective, inexpensive and non‐invasive method of distinguishing mostly between the sexes and breeds of cats but, not so effective in differentiation of ages (Riepert et al., 1996).

Calcaneus represents a solid bone that resists well to post‐mortem changes and is generally preserved undamaged in archaeological sites and in the fossil record (Riepert et al., 1996; Panciroli et al., 2017).

In this study, the authors obtained morphometric measurements from mediolateral radiographic images of the calcaneus in domestic cats. There are no osteological studies on the calcaneus bone in cats. One of the reasons for this is that large numbers of cadavers are required to collect statistically sufficient bone. It can take a long time to collect cadavers, especially in some cat breeds. However, with radiographic methods, more samples can be reached in a shorter time.

Despite this, it is thought that the findings of the study may be valuable to use as preliminary comparative information with the remains of mummified cats.

Polly (2010) reported that felines have a more elongated tuber calcanei compared to canines. This situation, Panciroli et al. (2017) explains with the fact that cats jump on the prey from an ambush position and need more ‘push’ force when running. In this study, although morphometrically different results were obtained between domestic cat breeds to a certain extent, it may be recommended to plan a study with a similar approach among owned or stray cats. Because of the differences that can be determined in the calcaneus, which is expected to be directly affected by living conditions, will provide an idea about the extent to which biomechanical factors can be directing.

In humans, as in animals, the most commonly fractured tarsal bone is the calcaneus. Measurement of calcaneal angle (Böhler angle) in human medicine helps diagnose intra‐articular fractures of calcaneus (Knight et al., 2006; Touissaint et al., 2013). It has been reported that a wider calcaneal angle accompanies the injured calcaneus (Schepers et al., 2007).

For these reasons, this angle, which is assumed to be clinically important, is thought to be important in obtaining reference values.

Human studies on calcaneus measurements were used to inform methodology of this study and the results could be compared with those of humans. In our study it was observed that the calcaneal angle values taken in cats were quite low (digitigrade posture animals) compared to humans (plantigrade) (Dyce et al., 2017). This angle that is 18.22 ± 1.97° in mix‐breed cats, 20.01 ± 2.42° in Scottish Folds and 18.6 ± 2.08° in British Shorthairs has been reported to be above 30° average in humans (Boyle et al., 2011; Rokaya et al., 2016; Seyahi et al., 2009).

Reference studies on the use of this angle in the field of clinical veterinary medicine have not been found. In this study, the differences of the calcaneal angle between sexes of cats were statistically examined, but no significant results were found. We think that the studies that will contribute clinically to veterinary surgery can be done with reference measurement taken from samples with calcaneal fractures.

In this study, as in the reference information, the linear measurements of male cats were found to be higher than those of females. Also, it is believed that it is necessary to expand the literature knowledge by doing more studies in the veterinary field. The data provided by this study of the radiological image measurements of the calcaneus in cats will be valuable because they can be used for other similar studies.

## AUTHOR CONTRIBUTIONS

Esra Şenol: conceptualisation; methodology; supervision; writing – original draft; writing – review & editing. Ozan Gündemir: conceptualisation; data curation; methodology; resources; software; writing – original draft; writing – review & editing. Sokol Duro: data curation; methodology; visualisation; writing – original draft; writing – review & editing. Tomasz Szara: formal analysis; methodology; software; writing – original draft; writing – review & editing. Yasin Demiraslan: methodology; resources; software; validation; writing – original draft; writing – review & editing. Hüseyin Karadağ: data curation; investigation; methodology; resources; validation; writing – original draft.

## CONFLICT OF INTEREST

The authors declare that they have no conflict of interest.

## FUNDING INFORMATION

This research did not receive any specific grant from funding agencies in the public, commercial, or not‐for profit sectors.

### ETHIC STATEMENTS

Permission Nr. 60618/16.03.2021 was obtained from the Ethics Committee of Istanbul University‐Cerrahpaşa, Faculty of Veterinary Medicine

### PEER REVIEW

The peer review history for this article is available at https://publons.com/publon/10.1002/vms3.899.

## Data Availability

The data that support the findings of this study are available from the corresponding author upon reasonable request.
